# Aids to Nursing

**Published:** 1888-03-03

**Authors:** M. M. Basil

**Affiliations:** Author of "The Commoner Accidents to Life and Limb"


### AIDS TO NURSING.

BY M. M. BASIL, M.A., M.B., C.M., Author of "The Commoner Accidents to Life and Limb."

(Continued from p. 364.)

### BED AND BEDDING.

WE shall consider next the bed and bedding of the sick-room. The bed must not be too high, in order that the patient may get into it and out of it easily, that his head may not be placed in a layer of vitiated atmosphere, and that he may be readily accessible to the nurse. The "lofty bed," associated from time immemorial with kings and such-like personages, is therefore a luxury not to be encroached upon by ordinary mortals. In certain cases, as in the treatment of epileptics, we are compelled to select a low bed to prevent accidents, from tumbling out during a fit. This is an arrangement, however, that must be avoided at all costs. The bedstead must be free from shakiness, especially in cases where the least movement would cause pain.

The bed must not be placed against a wall, except in rare cases, as in the treatment of delirious patients. It should be accessible from both sides, and the end should stand eight to twelve inches away from the nearest wall, so that free movement between the bed and the wall is possible. This requirement must not be sacrificed to any fad which may be entertained on the subject of supposed neatness, which the arrangement of beds right against the wall is thought to give to the ward. It should not be situated between the door and the fireplace, or a window and a fireplace, in order to prevent the exposure of the patient to the current of air which is certain to be established in such a position. It should be so placed as to avoid the exposure of the patient's face to a bright light.

A separate bed for the night is an excellent provision; you should therefore, in nursing private cases, try to secure two beds, with two sets of blankets, night dresses, etc. In a hospital such an arrangement is not possible, but a movable bed can always be had, or a couch or easy-chair and footstool may be extemporised into such a bed. In lifting a patient from one bed to another, please remember to place the bedsteads end to end, and not by the side of each other; you then lift the patient completely out of the first bed and over it into the second.

The best beds are rheocline spring or woven wire mattress ones. Horsehair is the best material for bedding; coire fibre is also excellent. Flock beds and feather beds must be avoided. In certain cases straw beds are of advantage. Thus, if there is persistent incontinence of urine or of fæces, comfortably-made straw beds will be very serviceable in public institutions. The mattress, instead of consisting of a single piece, should be made of three parts, accurately fitting each other when placed end to end; you can then change the centre piece frequently without disturbing the other portions. The best blankets to use are white Witney ones. The mattress and the bed covering should be as light as possible, so that free ventilation can be secured. In all conditions heavy quilts and counterpanes must be avoided; in serious disease, however, this question assumes a very important aspect. Hitherto it has not received the attention it deserves both at the hands of medical men and nurses. The Rev. Dr. Houghton, of Dublin, calculated that the work done by a man by the ordinary process of respiration alone, in the twenty-four hours, was equivalent to lifting eleven tons through a height of one foot. In many diseases, especially such as interfere with respiration primarily, there is no doubt that this work is increased largely, irrespective of any question of clothing. If we add to this the weight of the coverlets, which must be lifted up with each inspiration, we shall find that a large amount of muscular work is thrown upon a patient when in a position least fitted for such exertion. At present few would be inclined to run the risks of removing the weight of the bed covering entirely off the chest of such a patient by means of a bed-frame, and then supplying carefully-regulated artificial heat. Given, however, a nurse on whom one could place absolute confidence for conscientiousness and intelligence, such an arrangement would be beneficial to many patients.

It is important to remember that during severe frost spring-beds or beds with light mattresses are difficult to keep warm, because of the too free access of cold air to the patient from under the bed. This must be remedied by using additional mattresses. It is absurd to pile blankets on a patient when the cold air chills him from below. In district nursing you will find that paper makes a capital addition to your means of securing warmth. When blankets are insufficient, sheets of newspaper stitched on to the coverlets will often be of service.

Bed-frames, bed-rests, bed-baths, foot-warmers, and supports for the feet are necessary means for securing comfort, and will be considered as the indications for their use come under examination.

The bedstead ought to be systematically washed and cleaned, and the bedding thoroughly aired. Every bed ought to be aired, if possible, daily, and the mattress should undergo a thorough, honest shaking up once a week. Where the nature of the case under treatment will admit of the arrangement, it is best to have fixed days for "turning up" the beds. If the patient cannot leave the bed, it is necessary to ventilate the sheets from time to time. Artificial bed ventilation-tubes, such as O'Brien's, are often very useful. If the mattresses in use are thin, natural ventilation is more likely to be satisfactory.

Every case admitted into a hospital should have new sheets, and when death occurs the whole bedding must be changed.

Careful attention must be paid to the making of the sick-bed. Certain general rules can be laid down on the subject, provided you steadily remember that important minor variations must be made to suit the taste and peculiarities of each patient. Each individual must have the bed made according to his own style; the cut and the fashion of it must be considered here just as much as in the fit of his clothes. Some will have their pillows high, others low; some cannot rest until they have twisted and jammed and pommelled them to the right, and others to the left. It would be unwise to interfere with such peculiarities, on the absurd ground of securing neatness and uniformity in a ward. We do not live a single hour quite scientifically, and life would be intolerable if each of our peculiarities had to be checked to suit the precise rules of any and every body. The sick must be individualised. An attempt to follow uniformity in the management of everyone in a ward will produce restlessness, discontent, and that want of "home-like" feeling which is so often complained of in large institutions.

Badly-made beds are the cause of nearly all bed-sores; they have to answer for the majority of cases of sleeplessness, want of rest, and feverishness. The under-sheets must receive special attention. Every edge must be so well tucked in that it cannot possibly get loose or creased, however much the patient may roll about. This can be best secured by the use of safety-pins, of which the "unique patent" is perhaps the best. The sheets must be free from any creases, rucks, or seams. Sometimes a patient is expected to rest in a bed where the under-sheet have whole yards of rough seaming: a bed of thorns would be equally likely to suit a sick person. Crumbs of bread and portions of other food must be carefully kept out of the beds, but, in spite of all watchfulness, they will get entrance there, and a regular hunt after them must be instituted.

As a general rule, there should be no blankets under the patient. Some books make the sweeping assertion that you should *never* use under-blankets. It is well, however, to remember that where they are used the chances of having bed-sores are much increased. The blankets should not be heavy about the chest of the patient. If they are too long you should turn them up or in, mostly about the foot of the bed. If counterpanes are in use, you must be careful that no fringes can cause annoyance by coming in contact with the face of the patient. A similar precaution is necessary to prevent the hair of the blanket getting exposed and then tickling the patient.

The minutest attention to the cleanliness of the bedding is necessary in operation cases.

In nursing old and feeble patients you must be careful to cover the bed during the temporary absence of the invalid from it, as it takes a long time to regain the temperature comfortable to the patient.

The pillows should not be hard and lumpy. The proper way to "smooth the pillow" is a point that no nurse, however well trained in other respects, can afford to lose sight of. Changes in the position of the head may often be made, not directly, but from under the pillow.

At least two handkerchiefs should be placed under the pillows, as these articles, like the much-abused policemen, have a peculiar knack of disappearing under urgent circumstances.

If bed-jackets are worn, they should be provided with pockets.

The process of changing the bed-linen of a patient who is seriously ill is an operation of some delicacy, and requires careful consideration. You should select a time when the patient is least exhausted, as, for example, an hour after a meal. If the patient is getting stimulants, it is better to administer a dose about half-an-hour before the procedure. The thermometric reading of the room must also be ascertained. You get every necessary article ready before the patient is allowed to know of the intended change. There must be no attempt at dragging the patient either up or down or from side to side. In rare cases, as in the nursing of young people and of light women, changes of position in the bed may be effected by one nurse. You stoop over the bed, get one arm of the patient placed round your shoulders, then, supporting the patient's trunk well with your hands, lift him to the desired position. In all other cases it is necessary to have at least two nurses to change the position of a prostrate patient, and three nurses if an injured limb is to be attended to at the same time. To attempt changing the sheets single-handed is a gross blunder; its successful completion merits reprehension, and not recommendation.

When these preparatory steps have been settled the sheets may be changed quickly, either sideways or from one end of the bed to the other. In the first instance you divide the bed in the direction of its length into two halves. Roll the patient gently to one side; remove the soiled sheet from the unoccupied half, arrange the clean sheet there, then lift or roll the patient on to the portion where the clean sheet has been spread, while the soiled one, along with the unfolded part of the clean one, is withdrawn from under him. In certain cases, before lifting the patient, you may stitch one sheet to the other, so that they may be withdrawn more easily. This method is but slightly exhausting to the patient, and may be resorted to in nearly all serious medical cases. In the other procedure you change the sheets from top to bottom, or *vice versa*. In fractures of the lower extremity, in abdominal operations, and in other operations involving the trunk, it is often advisable to use the second method. The soiled sheet is removed, and a clean one spread on either the upper or lower end of the bed, then the patient is lifted up and the remaining portions of the soiled and clean sheets are simultaneously rolled up or down, as the case may be. The change of linen can often be effected without removing the blankets. *(To be continued.)*

